# Simultaneous presentation but contrasting clinical features in 2 female siblings with juvenile systemic lupus erythematosus (JSLE)

**DOI:** 10.1186/1546-0096-12-S1-P326

**Published:** 2014-09-17

**Authors:** Mahesh Janarthanan, Prahalad Nageswaran, Sathish Kumar

**Affiliations:** 1 Sri Ramachandra University, Chennai, India; 2 Mehta Children's Hospitals, Chennai, India; 3 Pediatric Nephrology, Mehta Children's Hospitals, Chennai, India; 4 Pediatrics, Mehta Children's Hospitals, Chennai, India

## Introduction

SLE is an autoimmune disease characterized by the presence of antinuclear antibodies and multisystem involvement. The presenting clinical features can be variable and the course of disease unpredictable. Studies have shown increased incidence of SLE in siblings of individuals with the disease.

## Objectives

We report 2 female siblings who presented with juvenile SLE simultaneously in May 2013 but had contrasting clinical features.

## Methods

Fifteen year old sibling 1 presented with history of low grade fevers since 2 months, oral ulcers, malar rash, alopecia, pallor, arthralgia, history of loss of weight and appetite. Investigations revealed anaemia, leukopenia, high ESR, and low C3, C4 levels. She also had ANA +ve 1: 1280, ds DNA +ve, lupus anticoagulant +ve, anticardiolipin antibody +ve, normal urine protein creatinine ratio and normal BP. She was treated with oral steroids, hydroxychloroquine, azathioprine and calcium supplements.

10 year old sibling 2 presented at the same time with history of high grade fever since one week with no obvious focus. She was initially worked up for infective cause and treated symptomatically. During second week of illness she developed rashes in her lower limbs with arthritis in her ankle joints. Within next few days she developed acute abdominal pain, pedal oedema, elevated blood pressure and seizures. Her investigations revealed anaemia , leukopenia, elevated ESR, low C3 C4, ANA +ve 1:1000,dsDNA positive, elevated urine protein and high BP of 160/110 mmHg. US abdomen showed mild ascites, CT angiogram of abdomen was normal and renal biopsy showed evidence of necrotising vasculitis. She was treated with intravenous steroids followed by oral steroids, IV cyclophosphamide, hydroxychloroquine, antihypertensives and antoconvulsants.

## Results

Two female siblings presented with contrasting clinical features of SLE at the same time. The elder sibling had a chronic history and presented with typical clinical features of SLE. The younger sibling presented with a very short history and aggressive clinical course. Genetic studies were not done as this is not available in our country.

## Conclusion

The occurrence of SLE in the 2 female siblings at the same time probably points to an environmental factor as a trigger in these two patients. The presenting clinical features however were in complete contrast and representative of the two ends of spectrum of the disease.

## Disclosure of interest

None declared.

